# A prospective clinical pilot study on the effects of a hydrogen peroxide mouthrinse on the intraoral viral load of SARS-CoV-2

**DOI:** 10.1007/s00784-020-03549-1

**Published:** 2020-09-02

**Authors:** Maximilian J. Gottsauner, Ioannis Michaelides, Barbara Schmidt, Konstantin J. Scholz, Wolfgang Buchalla, Matthias Widbiller, Florian Hitzenbichler, Tobias Ettl, Torsten E. Reichert, Christopher Bohr, Veronika Vielsmeier, Fabian Cieplik

**Affiliations:** 1 Department of Oral and Maxillofacial Surgery, University Hospital Regensburg, Regensburg, Germany; 2 Department of Otorhinolaryngology, University Hospital Regensburg, Regensburg, Germany; 3 Institute of Clinical Microbiology and Hygiene, University Hospital Regensburg, Regensburg, Germany; 4 Department of Conservative Dentistry and Periodontology, University Hospital Regensburg, Franz-Josef-Strauß-Allee 11, 93053 Regensburg, Germany; 5 Department of Infection Prevention and Infectious Diseases, University Hospital Regensburg, Regensburg, Germany

**Keywords:** SARS-CoV-2, COVID-19, Hydrogen peroxide, Mouthrinse, Gargle, Intraoral, Viral load

## Abstract

**Objectives:**

SARS-CoV-2 is mainly transmitted by inhalation of droplets and aerosols. This puts healthcare professionals from specialties with close patient contact at high risk of nosocomial infections with SARS-CoV-2. In this context, preprocedural mouthrinses with hydrogen peroxide have been recommended before conducting intraoral procedures. Therefore, the aim of this study was to investigate the effects of a 1% hydrogen peroxide mouthrinse on reducing the intraoral SARS-CoV-2 load.

**Methods:**

Twelve out of 98 initially screened hospitalized SARS-CoV-2-positive patients were included in this study. Intraoral viral load was determined by RT-PCR at baseline, whereupon patients had to gargle mouth and throat with 20 mL of 1% hydrogen peroxide for 30 s. After 30 min, a second examination of intraoral viral load was performed by RT-PCR. Furthermore, virus culture was performed for specimens exhibiting viral load of at least 10^3^ RNA copies/mL at baseline.

**Results:**

Ten out of the 12 initially included SARS-CoV-2-positive patients completed the study. The hydrogen peroxide mouthrinse led to no significant reduction of intraoral viral load. Replicating virus could only be determined from one baseline specimen.

**Conclusion:**

A 1% hydrogen peroxide mouthrinse does not reduce the intraoral viral load in SARS-CoV-2-positive subjects. However, virus culture did not yield any indication on the effects of the mouthrinse on the infectivity of the detected RNA copies.

**Clinical relevance:**

The recommendation of a preprocedural mouthrinse with hydrogen peroxide before intraoral procedures is questionable and thus should not be supported any longer, but strict infection prevention regimens are of paramount importance.

**Trial registration:**

German Clinical Trials Register (ref. DRKS00022484)

## Introduction

The coronavirus disease 2019 (COVID-2019), which is caused by a novel coronavirus SARS-CoV-2 (severe acute respiratory syndrome coronavirus 2), amounts to more than 19.9 million confirmed cases and more than 730,000 attributed deaths, as per August 11, 2020, and thus represents one of the greatest challenges for the whole healthcare sector in the twenty-first century [1].

The main routes of SARS-CoV-2 transmission are by direct contact or by airborne transmission due to inhalation of aerosols and respiratory droplets [2]. This puts healthcare professionals (HCPs) at high risk of nosocomial infection with SARS-CoV-2 [3], as it was already shown by a disproportionally high infection rate for HCPs in an early report from Wuhan, China [4]. Patients with SARS-CoV-2 infection exhibit a very high viral load in the oropharynx, the oral cavity, and the nose, irrespective of the presence of clinical symptoms [2, 5]. Therefore, particularly HCPs from specialties with close contact to this region such as dentists, maxillofacial surgeons, and otorhinolaryngologists are at tremendous risk of being infected and becoming potential carriers of the virus [6–13]. Accordingly, the first COVID-19-related fatality of a medical doctor documented globally was that of an otorhinolaryngologist in Wuhan on January 25, 2020 [14]. Furthermore, clinical procedures in these specialties and particularly in clinical dentistry often involve generation of aerosols [15], which further increases the risks of nosocomial infection with SARS-CoV-2 among HCPs [11].

Therefore, special infection control regimens have been introduced in dental practices all over the world, including patient triage, personal protective equipment (such as particulate respirators based on n-95 or FFP2 standards), use of rubber dam isolation, and general limitation of aerosol-generating procedures [7–9, 11, 16, 17]. Following a first recommendation by Peng et al. [12], some author groups also proposed to let patients perform preprocedural mouthrinses with oxidizing agents such as 1% hydrogen peroxide or 0.2% povidone iodine in order to reduce the intraoral viral load before conducting any intraoral procedures [7–9, 17, 18]. Accordingly, the American Dental Association suggested on their website on March 12, 2020, to have patients gargle with a 1% hydrogen peroxide solution before each appointment [19]. Likewise, this suggestion was also disseminated among German dentists by the Institut der Deutschen Zahnärzte in their brochure about recommended standard dental procedures during the COVID-19 pandemic from April 24, 2020 [20].

The recommendation of a preprocedural mouthrinse with hydrogen peroxide was mainly based on the general vulnerability of SARS-CoV-2 toward oxidation [12], and on the finding that products containing oxidizing agents such as hydrogen peroxide and povidone iodine were able to inactivate coronaviruses on inanimate surfaces within a 1-min exposure period [21, 22]. However, until now, there are no clinical data supporting the efficacy of suchlike preprocedural mouthrinses in terms of reducing the intraoral viral load in SARS-CoV-2-infected patients [23–26]. Due to the high viral load in the oropharynx and the oral cavity, there may be some recontamination soon after performing the mouthrinse [27]. Furthermore, caution should be taken before generally recommending antimicrobial mouthrinses due to the inherent risks of inducing detrimental shifts in the oral ecosystem [28].

Therefore, the aim of this clinical pilot study was to investigate the effects of a mouthrinse with 1% hydrogen peroxide on the intraoral viral load in SARS-CoV-2-positive patients.

## Material and methods

### Study design

The present study is a prospective clinical pilot study investigating the effects of a mouthrinse with 1% hydrogen peroxide on the intraoral viral load of SARS-CoV-2-positive patients hospitalized at the isolation ward of the University Hospital Regensburg during an investigation period of 2 months from April until May 2020.

Only patients with a positive test for SARS-CoV-2 within the last 72 h were included in this study. Exclusion criteria were indication for intubation or mechanical ventilation and severe stomatitis.

Patients were screened for eligibility by one medical doctor (IM) and provided with detailed description of the study outline which involved the following procedure: patients were asked to gargle their mouth and throat with 20 mL 0.9% NaCl for 30 s for acquiring a baseline oropharyngeal specimen for the SARS-CoV-2 real-time PCR (RT-PCR) test. Immediately afterwards, patients had to perform a mouthrinse with 20 mL 1% hydrogen peroxide by gargling their mouth and throat for 30 s. Thirty minutes after this mouthrinse, another oropharyngeal specimen for the SARS-CoV-2 RT-PCR test was acquired by letting the patients gargle their mouth and throat with 20 mL 0.9% NaCl for 30 s. The respective quantities of copies/mL of SARS-CoV-2 RNA were analyzed by RT-PCR.

Written informed consent was obtained from all individual participants included in the study. The study design was approved by the ethics committee of the University of Regensburg (ref. 20-1787-101) in accordance with the 1964 Helsinki declaration and its later amendments or comparable ethical standards. The study has been registered at the German Clinical Trials Register (ref. DRKS00022484).

### RT-PCR-based analysis of viral load

Nucleic acids were extracted from 200 μL of oropharyngeal specimens using EZ1 Virus Mini Kit v2.0 in combination with the EZ1 Advanced XL system (Qiagen, Hilden, Germany), as recommended by the manufacturer. Viral RNA was amplified and detected in duplicate using the SARS-CoV-2 E gene RT-PCR [29] on the StepOnePlus RT-PCR System (ThermoFisherScientific, Schwerte, Germany). Bacteriophage MS2 was used as internal control to check for extraction and amplification efficacy [30].

### Virus culture

SARS-CoV-2 was isolated from the oropharyngeal specimens that exhibited more than 10^3^ copies/mL of SARS-CoV-2 RNA at baseline by using kidney epithelial cells from African green monkey (Vero-CCL19 cells, ATCC). Cells were cultivated in Dulbecco’s Modified Eagle‘s Medium supplemented with 10% heat-inactivated fetal calf serum (Sigma-Aldrich, Munich, Germany), 90 U/mL streptomycin, 0.3 mg/mL glutamine, 200 U/mL penicillin, and 2.5 μg/mL amphotericin B (PAN Biotech, Aidenbach, Germany). After inoculation of oropharyngeal specimens for 24 h, cells were washed twice before viral loads in the supernatants were determined 7 days post-infection by RT-PCR as described above.

### Data analysis

Data are reported as median values (with 1st and 3rd quartiles) and were statistically analyzed non-parametrically using the Wilcoxon signed-rank test for related samples on a significance level of *α* = 0.05. All statistical analyses were performed using IBM SPSS Statistics, version 25 (IBM Corp., Armonk, NY, USA).

## Results

### Patient population

**Fig. 1:**
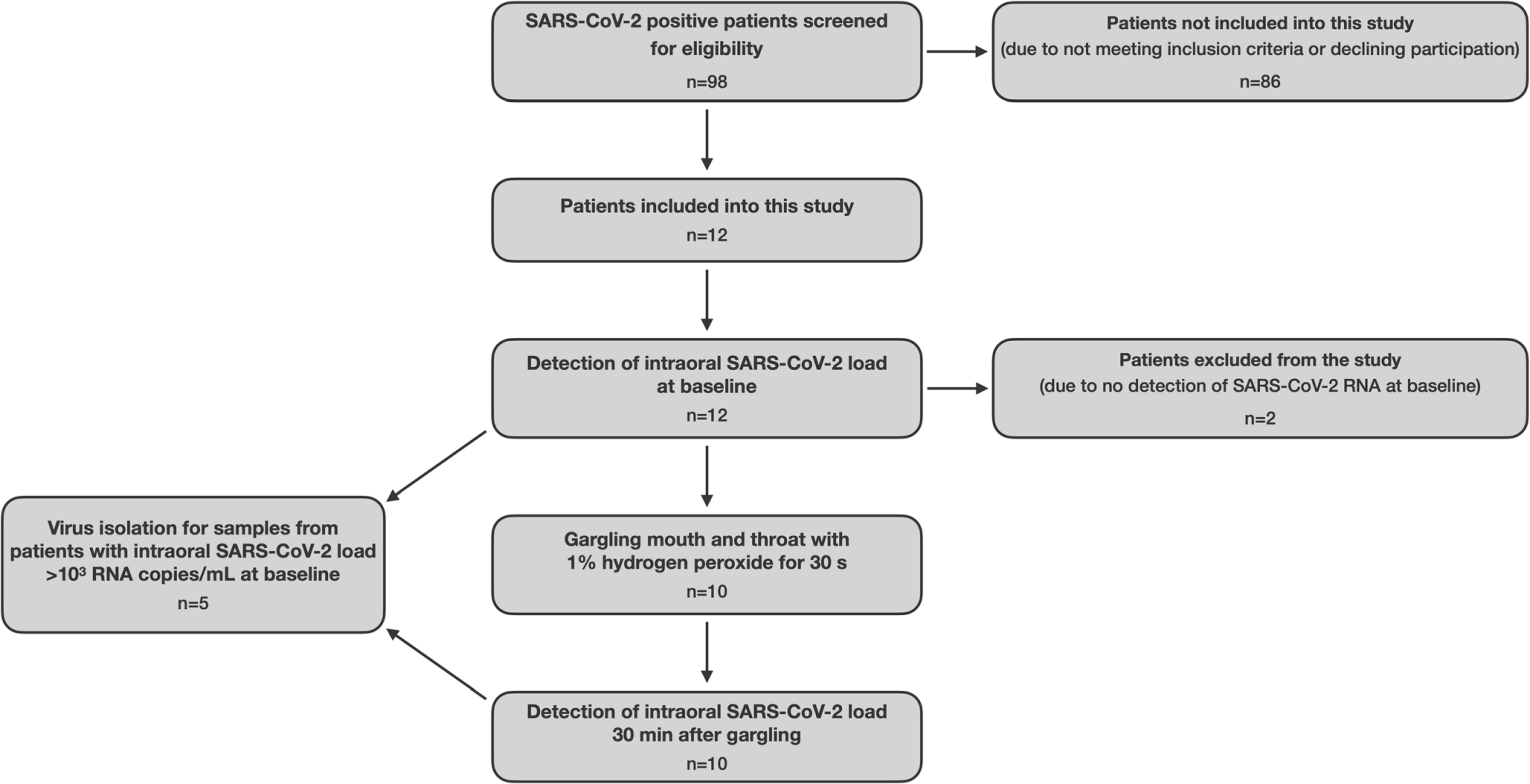
Flow of patients through the stages of this study

Twelve SARS-CoV-2-positive patients were included in this study. These 12 patients (6 female and 6 male) had a median age of 55 years (range: 22–81 years). One patient was hospitalized in an intensive care unit (without need of intubation), and 11 were hospitalized in an isolation ward. Eleven out of the 12 patients showed comorbidities (e.g., diseases of the liver, cardiovascular system or kidney, hematological diseases, and obesity). Only one of the patients presented no symptoms of the infection with SARS-CoV-2. The time period between diagnosis of SARS-CoV-2 infection and inclusion in the study ranged from 1 to 5 days (median 3 days). Table 1 shows a detailed overview of the patient population included in this study. Figure 1 shows the flow of patients through the stages of this study.

**Table 1:** Patient characteristics

Patient no.	Sex	Age	Admission symptoms	Underlying diseases	Period between diagnosis and study inclusion (days)
1*	w	60	Reduced general condition, cough, fever	Obesity, arterial hypertension	5
2	w	55	Fever, genitourinary infection	H/O liver transplant with immunosuppression	3
3	m	56	Loss of appetite	Cirrhosis of the liver	2
4	m	43	Reduced general condition, dyspnea	Granulomatosis with polyangiitis, H/O renal cell carcinoma	1
5	m	77	Cough, dyspnea	Chronic lymphocytic leukemia, multiple myeloma, arterial hypertension	1
6	w	57	Hydropic decompensation	Cirrhosis of the liver, coronary heart disease, depression, restless legs syndrome	3
7*	w	81	Tiredness	Anemia, H/O aortic valve replacement	4
8	w	47	Reduced general condition, cephalalgia, sore throat	Epilepsy	1
9	m	22	Reduced general condition, dyspnea	No underlying diseases	5
10	m	67	No symptoms. Positive in preoperative screening	Symptomatic coronary heart disease	4
11	w	39	Acute on chronic renal failure, exsiccosis	Chronic renal failure caused by familial primary hypomagnesemia with hypercalciuria and nephrocalcinosis	3
12	m	61	Reduced general condition, cough, fever	Chronic renal failure, arterial hypertension, coronary heart disease, obstructive sleep apnea, obesity	5

*These two patients were excluded from this study since no SARS-CoV-2 RNA could be detected in the baseline specimens prior to performing the 1% hydrogen peroxide mouthrinse

### RT-PCR-based analysis of viral load prior and after 1% hydrogen peroxide mouthrinse

In two out of the 12 initially included patients, no SARS-CoV-2 RNA could be detected in the baseline specimens prior to performing the 1% hydrogen peroxide mouthrinse. Therefore, these two patients were excluded from the study.

**Fig. 2:**
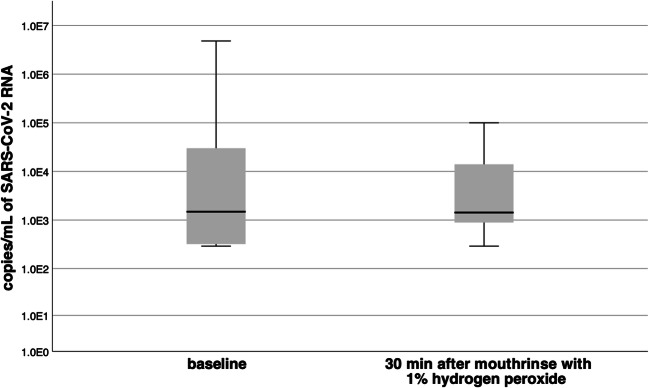
Copies/mL of SARS-CoV-2 RNA at baseline and 30 min after 1% hydrogen peroxide mouthrinse. All results are depicted as boxplots, which display medians, 1st and 3rd quartiles (box), minima and maxima (whiskers)

Table 2 shows the viral load of the remaining 10 individual patients at baseline and 30 min after the 1% hydrogen peroxide mouthrinse. The baseline specimens exhibited a median (1st; 3rd quartile) viral load of 1.8 × 10^3^ (3.1 × 10^2^; 4.7 × 10^4^) copies/mL of SARS-CoV-2 RNA. The second analysis 30 min after the 1% hydrogen peroxide mouthrinse showed a median (1st; 3rd quartile) viral load of 1.5 × 10^3^ (8.3 × 10^2^; 3.4 × 10^4^) copies/mL of SARS-CoV-2 RNA (Fig. 2). There were no significant differences between baseline viral load and viral load 30 min after the 1% hydrogen peroxide mouthrinse (*p* = 0.96).

**Table 2:** Intraoral viral load and virus culture

Patient no.	Baseline		30 min after gargling with 1% hydrogen peroxide mouthrinse	
	Copies/mL of SARS-CoV-2 RNA	Virus culture	Copies/mL of SARS-CoV-2 RNA	Virus culture
1*	0	/	/	/
2	5.7 × 10^2^	/	6.3 × 10^2^	/
3	7.9 × 10^2^	/	2.9 × 10^2^	/
4	2.9 × 10^2^	/	1.2 × 10^3^	/
5	9.6 × 10^4^	Negative	1.0 × 10^5^	Negative
6	2.8 × 10^3^	Negative	1.1 × 10^3^	Negative
7*	0	/	/	/
8	4.8 × 10^6^	Negative	9.3 × 10^4^	Negative
9	3.2 × 10^2^	/	8.9 × 10^2^	/
10	3.7 × 10^3^	Negative	1.4 × 10^4^	Negative
11	2.9 × 10^2^	/	1.7 × 10^3^	/
12	3.0 × 10^4^	Positive	3.7 × 10^3^	Negative

*These two patients were excluded from this study since no SARS-CoV-2 RNA could be detected in the baseline specimens prior to performing the 1% hydrogen peroxide mouthrinse

Solidus (/), not performed

### Virus culture

Virus culture was performed by infecting Vero cells and assessing the viral load in the supernatants 7 days post-infection for the specimens that exhibited more than 10^3^ copies/mL of SARS-CoV-2 RNA at baseline. Replicating virus could only be detected in one baseline specimen (Table 2).

## Discussion

The COVID-19 pandemic caused an exceptional situation where HCPs needed to develop infection control regimens and treatment strategies with very little scientifically supported data at hand, which led to clinical implementation of new concepts in a rapidity which was hardly ever seen in medical history [31]. In the fields of dentistry and maxillofacial surgery, preprocedural mouthrinses with hydrogen peroxide were recommended by American and German specialist societies in March and April 2020 for potentially reducing the intraoral viral SARS-CoV-2 burden before performing intraoral procedures [19, 20]. Interestingly, this recommendation was promptly implemented in clinical practice [12, 17, 25], despite lack of any clinical data [23, 24]. On the contrary, there was just data from one in vitro study showing that a product containing 0.5% “accelerated” hydrogen peroxide (Virox^®^ Technologies Inc., Oakville Canada) was able to readily inactivate coronaviruses on inanimate surfaces within 1 min [21, 22, 26, 32]. Accordingly, the rapid development of clinical trials on the efficacy of suchlike preprocedural mouthrinses was strongly encouraged in a recent literature review [23]. Therefore, this study aimed to investigate the effects of a 1% hydrogen peroxide mouthrinse on the intraoral viral load in SARS-CoV-2-positive subjects.

This pilot study comprised 10 SARS-CoV-2-positive subjects, who served as their own controls. Therefore, no additional control group (e.g., using a placebo mouthrinse without hydrogen peroxide) was deemed necessary for this pilot study. The intraoral viral load of each patient was examined at baseline and after gargling with 20 mL 1% hydrogen peroxide for 30 s. For sampling, oropharyngeal specimens collected by mouth and throat rinses were chosen because recent studies showed that these specimens contain a higher viral load than nasopharyngeal or oropharyngeal swabs [33], and thus may also improve accuracy of SARS-CoV-2 detection [34]. The patients themselves performed the sampling under the supervision of a HCP without any need of invasive procedures, thus reducing the risk of nosocomial SARS-CoV-2 transmission to HCPs [34, 35].

This study revealed that a 1% hydrogen peroxide mouthrinse had no effect on reducing the intraoral viral load in SARS-CoV-2-positive subjects. Therefore, any mechanical effects due to the mouthrinse itself irrespective of its ingredients can be excluded. As RT-PCR-based analysis is only able to detect RNA copies but cannot give any indication on the infectivity of the detected virus fragments, we further tried to culture SARS-CoV-2 virus from the patients whose baseline specimens exhibited a viral load of at least 10^3^ RNA copies per mL. These specimens were used for infecting Vero cells, and the viral load was determined in the supernatants 7 days post-infection. Here, active virus replication could only be detected from one baseline specimen, thus not allowing conclusions on the effects of hydrogen peroxide on SARS-CoV-2 infectivity. Wölfel et al. showed that virus culture depends on viral load with specimens containing less than 10^6^ RNA copies per mL hardly yielding successful culture [36]. Therefore, the rather low median viral load of 1.8 × 10^3^ RNA copies/mL in the baseline specimens may be accounted for the absence of active virus replication after infection of cell cultures in most specimens of the present study. Likewise, To et al. found active virus in only 3 out of 12 saliva specimens from SARS-CoV-2-positive patients despite that the median viral load in these specimens was markedly higher (3.3 × 10^6^ RNA copies/mL) than in the present study [35]. The rather low median numbers of RNA copies per mL found in this study may mainly be due to the exclusion of patients with indication for intubation or mechanical ventilation, because it is well-known that the oropharyngeal and nasopharyngeal SARS-CoV-2 viral load strongly correlates with COVID-19 severity [24, 37]. On the other hand, inclusion of hospitalized patients with rather mild symptoms of COVID-19 like in the present study may rather represent the patient population of interest for the scope of this study. Asymptomatic patients or patients with mild symptoms, who are not aware that they are SARS-CoV-2 positive, may tend to visit dental, maxillofacial, or otorhinolaryngological practices more often than those with severe symptoms.

Despite the small number of patients included in this pilot study, the results reported here clearly show that gargling mouth and throat with a 1% hydrogen peroxide mouthrinse for 30 s does not decrease the intraoral viral load in SARS-CoV-2-positive subjects. In a very recent in vitro study, Bidra et al. investigated the virucidal effects of 1.5% and 3.0% hydrogen peroxide and 1.0%, 2.5% and 3.0% povidone iodine toward SARS-CoV-2 [38]. They incubated the test compounds with virus solution for 15 or 30 s, respectively, and then conducted a standard end-point dilution assay by plating the serially diluted surviving virus on Vero 76 cells. Presence or absence of cytopathic effects was assessed after incubation for 5 days. They found that 1.5% and 3.0% hydrogen peroxide had only minimal virucidal activity toward SARS-CoV-2, whereas povidone iodine led to the complete inactivation of SARS-CoV-2 at the lowest concentration of 0.5% and the lowest contact time of 15 s [38]. Based on this in vitro data, further studies should investigate the effects of povidone iodine mouthrinses on the intraoral viral load in SARS-CoV-2-positive subjects [24].

In view of the results of this clinical pilot study and the in vitro results reported by Bidra et al. [38], the recommendation of a preprocedural mouthrinse with hydrogen peroxide before any intraoral procedures is questionable and thus should not be supported any longer. In this context, the impact of a “false sense of security” in HCPs due to the hydrogen peroxide mouthrinse and consequent unfocused treatment of potentially infectious patients should be considered. Therefore, strict infection prevention regimens concerning infection of HCPs are of paramount importance until further studies are available.

## Conclusion

This pilot study shows that gargling mouth and throat with 1% hydrogen peroxide does not decrease the intraoral viral load in SARS-CoV-2-positive subjects. Further studies should investigate preprocedural mouthrinses containing other agents like povidone iodine for reducing the intraoral SARS-CoV-2 load. In the meantime, risk stratification by patient triage, obtaining a detailed anamnesis, providing personal protective equipment, and preventing the formation of droplets and aerosols during the treatment of patients appear to represent the most reliable infection control regimens for HCPs until further studies are available.
